# Season of birth and sugary beverages are predictors of Raven’s Standard Progressive Matrices Scores in adolescents

**DOI:** 10.1038/s41598-020-63089-2

**Published:** 2020-04-09

**Authors:** Reem Al-Sabah, Abdullah Al-Taiar, Abdur Rahman, Lemia Shaban, Anwar Al-Harbi, Olusegun Mojiminiyi

**Affiliations:** 1 Department of Community Medicine and Behavioural Sciences, Faculty of Medicine, Kuwait University, Box: 24923, Safat, 13110 Kuwait; 2 School of Community & Environmental Health, College of Health Sciences, Old Dominion University, 4608 Hampton Blvd, Norfolk, VA 23508 US; 3 Department of Food Science and Nutrition, College of Life Sciences, Kuwait University, Box 5969, Safat, 13060 Kuwait; 4 Department of Pathology, Faculty of Medicine, Kuwait University, Box: 24923, Safat, 13110 Kuwait

**Keywords:** Health sciences, Risk factors

## Abstract

To investigate factors associated with cognitive functioning in healthy adolescents, a school-based cross-sectional study was conducted on 1370 adolescents aged 11–16 years that were randomly selected from all governorates of Kuwait. Raven’s Standard Progressive Matrices (SPM), a non-verbal test of intelligence, was used to measure cognitive functioning of the study participants. Data on predictors of cognitive functioning were collected from parents and adolescents. Weight and height of the participants were measured in a standardized manner and blood samples were tested in an accredited laboratory under strict measures of quality control. In multivariable linear regression analysis, factors that showed significant association with the SPM score were gender (p = 0.002), season of birth (p = 0.009), place of residence (p < 0.001), father’s (p < 0.001) and mother’s (p = 0.025) educational level, type of housing (p < 0.001), passive smoking at home (p = 0.031), sleeping hours during weekends (p = 0.017), students’ educational level (p < 0.001) and the frequency of consumption of sugary drinks (p < 0.001). The link between cognitive functioning and season of birth seems to be robust in various geographical locations including the Middle East. The association between sugary drinks and cognitive functioning highlights the importance of diet independently of obesity and support efforts to reduce consumption of sugary drinks among children.

## Introduction

Cognitive function comprises various mental processes such as attention, memory, and language that are required to manage daily life activities in all age groups. Among children and adolescents, cognitive functions are essential for good school performance and educational achievements. More importantly, cognitive function during childhood and adolescence is strongly linked to various health outcomes in adulthood. As an example, in adulthood, children with poor cognitive functions have an increased risk of hypertension^1^, coronary heart diseases and stroke^2^, psychological disorders^3^, and all-cause mortality^4–6^. These associations seem to be strong, incremental across the whole spectrum of cognitive function and independent of socioeconomic conditions^7^.

Much of the recent public health research has focused on factors associated with the decline of cognitive function among the elderly. This is understandable because populations are aging in most countries, and the decline of cognitive function has become a major public health issue. Additionally, there has been substantial research on factors associated with the development of cognitive functions during early life, but cognitive abilities among healthy school-aged children have hardly been addressed in the literature. This holds true in spite of the fact that some areas of the human brain as well as higher cognitive functions continue to develop during adolescents^8^.

Predictors of cognitive function have also been thoroughly investigated among children and adolescents with various disease conditions such as children with specific syndromes^9,10^, attention-deficit hyperactivity disorder^11^, sickle cell anemia^12^, beta-thalassemia^13^, HIV^14,15^, epilepsy^16^, type 1 diabetes^17,18^ and many other disease conditions. Among healthy adolescents, studies have focused on the role of poverty and undernutrition^19^, iron deficiency and parasitic infections^20^, parental education^21^, or overall socioeconomic status^22^. These studies were mainly in low-income countries and thus their findings cannot be extrapolated to high-income settings such as the oil-rich Arab countries in the Middle East. In these countries, there has been a dramatic change in socioeconomic status in the last few decades, which has eliminated poverty and undernutrition and led to significant improvements in education. Therefore, factors like poverty and undernutrition are unlikely to be a major predictor of cognitive function among children in this setting. In these settings, new factors may have emerged such as overweight and obesity in addition to sedentary lifestyle, which have been linked to poor cognitive function^23^. We have previously reported that vitamin D levels is not associated with cognitive function^24^. In this study, we aim to investigate factors associated with cognitive functioning as measured by non-verbal test of intelligence, including obesity, physical activity, and nutritional deficiencies such as low levels of vitamin B_12_, iron and ferritin.

## Methods

Kuwait is a small country with a population of 4.2 million and is divided into six provinces (governorates). A school-based cross-sectional study was conducted on students in middle schools typically aged 11–16 years. The details of the project have been published previously^24,25^. In brief, we recruited students from 12 public middle schools that were randomly selected using stratified multistage cluster random sampling with a probability proportional to size from all governorates of Kuwait. In each governorate, schools with larger number of students were given a higher probability of being selected compared to school with small number of students. The sample allocation in each governorate was based on the relative size of that province as judged by the total number of students in the governorate. The study was funded by Kuwait University and approved by The Ethics Committee at the Ministry of Health in Kuwait (No: 2015/248) as well as The Ethics Committee at the Health Sciences Centre, Kuwait University (No: DR/EC/2338). The study was conducted in accordance with Declaration of Helsinki ethical principles for medical research involving human subjects. An informed consent was taken from the parents of each study participant.

### Collecting data on predictors of cognitive functioning

An informed consent was sent to the parents along with a self-administered questionnaire on parental level of education and income, type of housing, number of siblings of the index child, passive smoking in the household and the number of times per week the index child had a meal prepared outside the home during the last three months. After obtaining written informed consent from the parents and verbal assent from the school children, trained dedicated personnel carried out face-to-face interviews with the students using a structured questionnaire. The questionnaire was carefully developed after an extensive review of the literature and was pilot tested on 20 students who were not included in the study. We included questions on physical activity utilizing a questionnaire that was developed based on the Youth Physical Activity Questionnaire in UK^26^ and The Arab teens lifestyle study^27^. This questionnaire was validated among high school students and showed a strong correlation with data collected using accelerometers (Spearman correlation 0.92; p < 0.001 for total steps count) (not published). Anthropometric measurements, including standing height and body weight of the study subjects, were assessed using a digital weight and height scale in a standardized manner.

Blood sample of five ml of venous blood was drawn from each participant by a trained nurse. On the same day, the samples were centrifuged, and the serum was transferred to Eppendorf tubes and stored at −80 °C for analysis. In these blood samples vitamin D was measured using liquid chromatography-tandem mass spectrometry (LC-MS/MS). Complete blood count (CBC), Parathyroid Hormone (PTH), vitamin B_12_, Iron, ferritin, transferrin and transferrin saturation were all measured in an accredited Teaching Hospital Clinical Biochemistry laboratory, where these tests are routinely conducted under strict quality control. Lead in whole blood (50 µL) was measured using ESA’s LeadCare II® Blood Lead Testing System (RNA Medical Bionostics, Inc. Devens, MA, USA) according the manufacturer’s instructions. Because this method was deemed to be not accurate, lead was evaluated again in all blood samples using Inductively Coupled Plasma Mass Spectrometry (ICP-MS).

### Assessment of cognitive functioning

Comprehensive assessment of cognitive function requires using several tests that evaluate verbal cognition, non-verbal cognition, executive functioning and processing speed. However, conducting such comprehensive assessment in large epidemiological studies in school settings would be logistically difficult. Furthermore, many cognitive tests are heavily influenced by language and culture. We used Raven’s Standard Progressive Matrices (SPM) to assess the cognitive functioning of the study participants. The test is culturally fair and non-verbal measure of intelligence (mainly fluid intelligence) and measures abstract reasoning, problem solving, perceptual awareness and reasoning by analogy. It measures participant’s eductive (to make meaning out of chaos), as opposed to reproductive (to understand and recall previously learned information) ability^28^. In SPM, participants are asked to identify the missing piece of an array of patterns by determining which rule or rules govern the patterns displayed in the rows and columns. SPM has been previously validated in our setting^29^. In fact, SPM is used routinely to measure cognitive function in schools as part of the psychological and social services provide by The Ministry of Education in Kuwait. Specialists from The Department of Psychological and Social Services at The Ministry of Education administered SPM in the schools. Raw scores were converted to standard scores as per the instruction manual^30^. We will use the term cognitive function or cognitive functioning to refer to results of the Raven’s SPM in our study.

### Sample size estimation and power calculation

The required sample size was estimated using the Stata command *powerreg* to provide more than 90% power to detect a change of 0.03 in the coefficient of determination (R^2^) at 5% level of significance assuming that R^2^ in the full model is 0.20 taking into account the number of total predictors included in the study (29 predictors in total and 64 dummy variables). While the calculation showed that 1131 study subjects would be sufficient, an addition of 20% of the calculated sample size was added to compensate for missing data.

### Statistical methods

Data were double entered into specifically designed database using Epidata Entry. Data analysis was conducted using Stata version 12 (StataCorp. College Station, TX, USA). BMI was calculated as weight (Kg) divided by height squared (m^2^), which was later categorized into normal, overweight and obese according to WHO growth charts. The main outcome in the analysis was SPM score, which was approximately normally distributed; hence we used linear regression models to investigate the predictors of SPM score. Guided by the conceptual frameworks in the literature^31,32^, variables were divided into groups. The first group included socio-demographic factors such as sex, nationality, parental education, total number of siblings, father’s income, mother’s employment, type of housing, having private room for the index child in the house, child rank and whether the child lives with both parents in addition to season of birth. The second group included educational level of the participant, hours of sleep during weekdays and weekends, whether the child takes a nap during the day at weekdays and weekends, whether the child is currently taking vitamins or supplements, and frequency of sugary drinks consumption. The third group included total time of physical activity and BMI categories as per WHO growth charts; while the fourth group included vitamin D, parathyroid hormone (PTH), hemoglobin (Hb), calcium, iron, ferritin, transferrin and transferrin saturation in addition to lead. Continuous variables were fitted either as continuous or categorized using acceptable cutoff points or using tertiles. In univariable analysis, variables that showed significant association with SPM score at a conservative α level < 0.2 were included in multivariate analysis. Then variables in each group were added sequentially to the model and those with p < 0.05 were retained in the model. Interaction terms were fitted in the model when there is a prior knowledge that support presence of the interaction. The assumptions for this analysis were checked including the linear relationship assumption using scatter plots and partial regression plots. Homoscedasticity assumption was checked by Breusch-Pagan test and by plotting the studentized residuals against the unstandardized predicted values. Multicollinearity was checked by the inspection of correlation coefficients matrix between independent variables and variance inflation factor. Normality of the residuals was checked by Q-Q plot. Finally, we used stepwise backward and forward selection approaches to identify factors that were independently associated with the cognitive function as a confirmatory method.

## Results

Of the 1583 parents approached, 161 refused to participate (either child or parents refused). Another 6 adolescents had samples that were not sufficient to conduct blood analysis. Of the remaining 1416 adolescents, 1370 completed the SPM and their socio-demographic characteristics are shown in Table 1. Table 2 shows the association between various factors presumed to be associated with SPM scores in univariable analysis. Most socio-economic factors showed associations with SPM scores in univariable analysis. However, except for PTH and calcium, most of the laboratory measurements such as hemoglobin, iron, ferritin, transferrin, transferring saturation and vitamin D level were all not associated with SPM scores. In multivariable analysis, factors that showed significant associations with SPM scores were gender (p = 0.002), season of birth (p = 0.009), place of residence (province) (p < 0.001), father’s (p < 0.001) and mother’s (p = 0.025) educational level, type of housing (p < 0.001), passive smoking at home (p = 0.031), sleeping hours during weekends (p = 0.017), students’ educational level (grade) (p < 0.001) and the frequency of consumption of sugary drinks (p < 0.001) (Table 3). Both backward and forward stepwise regression selected the same variables and these variables were also the same as the manually developed model above. However, PTH as a continuous variable was significant in the stepwise selection (β = −0.27; p = 0.04) but not in the manually developed model (β = −0.23; p = 0.065). It is worth noting that PTH (when fitted as a categorical variable using quartiles or using clinically acceptable cutoff points) was not significantly associated with SPM neither in the stepwise selection nor in the manually developed model.

**Table 1:** Socio-demographic characteristics of 1370 adolescents in public middle schools in Kuwait.

Characteristics		
Age in years, Mean (SD) years	12.40	(0.93)
	n	(%)
**Gender**		
Male	674	(49.2)
**Nationality**		
Kuwaiti	1,047	(76.42)
Non-Kuwait	323	(23.58)
Father’s Education^a^		
No formal education	15	(1.12)
Primary/Intermediate	215	(16.08)
Secondary (high school)	330	(24.68)
Diploma	251	(18.77)
University & above	526	(39.34)
Mother’s Education^**b**^		
No formal education	31	(2.30)
Primary/Intermediate	145	(10.74)
Secondary (high school)	294	(21.78)
Diploma	293	(21.70)
University & above	587	(43.48)
**Father’s Income**^**c**^ **(Kuwaiti Dinars)**		
Less than 500	89	(6.72)
500 to 1000	291	(21.98)
1001 to 1500	414	(31.27)
1501 to 2000	213	(16.09)
More than 2000	164	(12.39)
Do not wish to tell	153	(11.56)
**Mother’s Employment Status**^**d**^		
Housewife	466	(34.72)
Paid employment	664	(49.48)
Others	212	(15.80)
**Housing**^**e**^		
Rented flat	499	(36.94)
Rented house	159	(11.77)
Owned flat	55	(4.07)
Owned house	638	(47.22)

^a^Missing for 33 participants; ^b^Missing for 20 participants; ^c^Missing for 46 participants; ^d^Missing for 28 participants; ^e^Missing for 19 participants.

**Table 2:** Association between Cognitive Function (Raven’s Progressive Matrices scores) and various factors among adolescents in univariable analysis.

Variables	β	(95% CI)	p-value^a^
**Gender**			
Male		[Reference]	0.120
Female	1.97	[−0.51,4.45]	
**Nationality**			
Kuwaiti		[Reference]	0.090
Non-Kuwaiti	2.52	[−0.40,5.45]	
**Season of birth**			
Summer (May, June, July)		[Reference]	0.045
Fall (August, September, October)	-3.40	[−6.92,0.13]	
Winter (November, December, January)	-4.70	[−8.18, −1.23]	
Spring (February, March, April)	−3.94	[−7.52, −0.36]	
**Province**			
Capital		[Reference]	<0.001
Hawally	−0.41	[−5.00,4.19]	
Farawanya	−11.80	[−16.48, −7.12]	
Jahra	−14.96	[−19.62, −10.30]	
Mubarak al-Kabeer	−1.38	[−6.54,3.78]	
Ahmadi	0.18	[−4.11,4.48]	
**Father’s education**			
Intermediate school or below		[Reference]	<0.001
Secondary (high-school)	1.90	[−1.92,5.73]	
Diploma	7.05	[2.99,11.12]	
University and above	13.31	[9.79,16.83]	
**Mother’s education**			
Intermediate school or below		[Reference]	<0.001
Secondary (high-school)	4.37	[0.11,8.62]	
Diploma	8.23	[3.98,12.49]	
University and above	14.09	[10.26,17.93]	
**Number of siblings**			
≤2		[Reference]	<0.001
3–4	−3.99	[−7.30, −0.69]	
≥5	−8.86	[−12.15, −5.57]	
**Father income**			
Less than 500 KD		[Reference]	
500–1000 KD	−2.66	[−8.17,2.84]	<0.001
1001–1500 KD	−8.35	[−13.66, −3.05]	
1501–2000 KD	−2.50	[−8.24,3.23]	
More than 2000KD	0.27	[−5.71,6.25]	
Do not wish to tell	−4.16	[−10.22,1.89]	
**Mother employment**			0.010
Housewife		[Reference]	
Paid employment	4.25	[1.50,7.01]	
Other	2.56	[−1.21,6.34]	
**Type of housing**			<0.001
Rented flat		[Reference]	
Rented house	−15.48	[−19.54, −11.41]	
Owned flat	−4.44	[−10.80,1.90]	
Owned house	−7.70	[−10.37, −5.03]	
**Having private room at home**			
Yes		[Reference]	0.027
No	2.83	[0.32,5.34]	
**Child birth order (Rank)**			
First		[Reference]	0.007
Second	−1.71	[−5.37,1.97]	
Third	−1.21	[−5.06,2.63]	
Fourth	−3.29	[−7.29,0.71]	
Fifth or above	−6.76	[−10.51, −3.02]	
**Passive smoking at home (Cigarettes)**			
No		[Reference]	<0.001
Yes	−4.98	[−7.59, −2.37]	
**Passive smoking at home (Shesha)**			
No		[Reference]	0.081
Yes	−4.69	[−9.97,0.58]	
**Frequency of consumption of breakfast prepared outside home**			
Never or almost never		[Reference]	0.014
1–2 times per week	−2.19	[−4.89,0.52]	
3–4 times per week	−7.22	[−11.95, −2.48]	
5 or more times per week	−4.46	[−9.91,0.99]	
**Frequency of consumption of lunch prepared outside home**			
Never or almost never		[Reference]	0.002
1–2 times per week	4.15	[1.23,7.08]	
3–4 times per week	0.96	[−3.98,5.90]	
5 or more times per week	−4.52	[−10.46,1.43]	
**Frequency of consumption of dinner prepared outside home**			
Never or almost never		[Reference]	0.067
1–2 times per week	−1.46	[−5.59,2.66]	
3–4 times per week	−5.19	[−10.00, −0.38]	
5 or more times per week	−4.63	[−10.68,1.43]	
**Educational level (grade in the school)**			0.032
Six		[Reference]	
Seventh	2.50	[−0.46,5.45]	
Eights	3.90	[0.86,6.93]	
**Family member help in study subjects**^**b**^			
No		[Reference]	<0.001
Yes, regularly	−11.90	[−15.46, −8.35]	
Yes, sometimes	−3.04	[−6.41,0.33]	
Yes, rarely	−2.80	[−7.90,2.29]	
**Having personal tutor to help in any study subject** ^**b**^			
No		[Reference]	<0.001
Yes	−6.76	[−9.27, −4.29]	
**Sleep during the weekdays (self-reported)**			
First tertile (<7.5 hours)		[Reference]	0.474
Second tertile (7.5 to <9 hours)	1.90	[−1.16,4.96]	
Third tertile (≥ 9 hours)	0.86	[−2.17,3.90]	
**Sleeping nap during the day at weekdays**			
No		[Reference]	0.168
Yes	−1.75	[−4.24,0.74]	
**Sleep during the weekends (self-reported)**			
First tertile (<9 hours)		[Reference]	0.042
Second tertile (9 to <11 hours)	0.35	[−2.92,3.62]	
Third tertile (≥ 11 hours)	−3.08	[−6.45,0.28]	
**Sleeping nap during the day at weekends**			
No		[Reference]	0.003
Yes	−6.8	[−11.22, −2.39]	
**Having medical condition that limits physical activities (self-reported)**			
No		[Reference]	0.022
Yes	−4.54	[−8.43, −0.66]	
**Time of the first meal during weekdays**			
Before 7 am		[Reference]	0.033
7–8 am	3.06	[−1.62,7.75]	
8–9 am	11.97	[3.43,20.51]	
9–10 am	1.74	[−1.06,4.55]	
After 10 am	4.30	[−1.30,9.89]	
**Time of the first meal during weekends**			
Before 7 am		[Reference]	0.100
7–8 am	8.91	[−2.38,20.20]	
8–9 am	9.29	[−0.97,19.55]	
9–10 am	10.22	[0.47,19.96]	
After 10 am	6.87	[−2.62,16.37]	
**Consumptions of sugary drinks per week**			
Zero time		[Reference]	<0.001
One per week times	−7.77	[−12.32, −3.22]	
Two per week times	−6.36	[−10.72, −2.00]	
Three per week times	−9.47	[−14.19, −4.76]	
Four to six times	−11.64	[−16.63, −6.65]	
Seven times or more	−15.28	[−19.68, −10.88]	
**Currently taking vitamins or supplements**			
No		[Reference]	0.671
Yes	−0.86	[−4.84,3.12]	
**Total time of physical activity**			
First tertile (Low)		[Reference]	0.903
Second tertile (Middle)	0.20	[−2.87,3.27]	
Third tertile (High)	0.68	[−2.39,3.75]	
**Body mass index as per WHO growth Charts**			
Obesity		[Reference]	0.100
Overweight	2.65	[−0.73,6.03]	
Normal	1.32	[−1.55,4.19]	
Severe thinness/Thinness	−8.69	[−18.71,1.32]	
**Hemoglobin level (as per WHO cutoffs)**			
Not anemic		[Reference]	
Anemic	−1.02	[−5.65,3.61]	0.665
**Iron** (umol/L)	−0.01	[−0.240,0.22]	0.935
**Ferritin** (ng/mL)	0.01	[−0.06,0.08]	0.827
**Transferrin** (g/L)	−0.10	[−3.44,3.24]	0.953
**Transferrin saturation** (%)	−0.02	[−0.18,0.13]	0.753
**Vitamin D (**nmol/L**)**	0.03	[−0.02,0.08]	0.234
**Parathyroid Hormone** (pmol/L)	−0.33	[−0.59, −0.07]	0.011
**Vitamin B**_**12**_ (pmol/L)	−0.03	[−0.08,0.02]	0.280
**Calcium** (mmol/L)	12.88	[−0.50,26.27]	0.059
**Lead** (5 ug/dL)	0.04	[−0.16–0.24]	0.684

^**a**^p-values were generated by partial F-test. ^b^These factors were excluded from multivariate analysis because they were deemed to be a result of low cognitive functioning.

**Table 3:** Factors associated with cognitive function (Raven’s Progressive Matrices scores) in multivariable analysis.

Variables	β	(95% CI)	p-value^a^
**Gender**			
Male		[Reference]	0.002
Female	3.92	[1.46,6.38]	
Season of birth			
Summer (May, June, July)		[Reference]	0.009
Fall (August, September, October)	−3.81	[−7.16, −0.46]	
Winter (November, December, January)	−4.87	[−8.19, −1.56]	
Spring (February, March, April)	−5.24	[−8.64, −1.84]	
**Province**			
Capital		[Reference]	<0.001
Hawally	0.76	[−3.73,5.26]	
Farawanya	−9.97	[−14.61, −5.32]	
Jahra	−6.07	[−11.08, −1.06]	
Mubarak al-Kabeer	1.69	[−3.38,6.77]	
Ahmadi	−0.83	[−5.18,3.52]	
**Father’s education**			
Intermediate school or below		[Reference]	<0.001
Secondary (high-school)	−0.18	[−4.01,3.65]	
Diploma	1.93	[−2.27,6.13]	
University and above	6.27	[2.42,10.12]	
**Mother’s education**			
Intermediate school or below		[Reference]	0.025
Secondary (high-school)	1.89	[−2.45,6.22]	
Diploma	1.37	[−3.13,5.87]	
University and above	5.19	[0.96,9.43]	
Type of housing			<0.001
Rented flat		[Reference]	
Rented house	−7.93	[−12.22, −3.63]	
Owned flat	−1.94	[−8.24,4.36]	
Owned house	−4.64	[−7.42, −1.85]	
Cigarettes smoking at home			
No		[Reference]	
Yes	−2.79	[−5.34, −0.25]	0.031
**Sleep hours during the weekends**			
First tertile (<9 hours)		[Reference]	0.017
Second tertile (9 to<11 hours)	0.10	[−2.94,3.33]	
Third tertile (≥ 11 hours)	−3.49	[−6.76, −0.22]	
**Grade level**			
Six		[Reference]	<0.001
Seventh	3.17	[0.32,6.03]	
Eights	5.60	[2.68,8.54]	
**Consumptions of soft drinks per week**			
Zero time		[Reference]	<0.001
One per week times	−6.46	[−10.81, −2.10]	
Two per week times	−3.60	[−7.77,0.56]	
Three per week times	−6.24	[−10.81, −1.67]	
Four to six times	−5.93	[10.87, −1.00]	
Seven times or more	−9.92	[−14.30, −5.54]	
**Parathyroid Hormone** (pmol/L)	−0.23	[−0.47,0.01]	0.065

^a^p-values were generated by partial F-test.

## Discussion

Poor cognitive functioning in children and adolescents is extremely important not only because it affects school performance and academic achievements but also predicts various disease conditions in adulthood^2–5^. In this study we aimed to investigate factors associated with cognitive functioning, as measured by a non-verbal test of intelligence (Raven’s SPM), among adolescents in a high-income setting in Kuwait. Socio-demographic factors, season of birth and consumption of soft sugary drinks were the main predictors of cognitive functioning while obesity and laboratory markers such as Hb, iron, ferritin, calcium and vitamin B_12_ were all not significantly associated with cognitive functioning among adolescents.

With the ever-increasing world-wide prevalence of childhood obesity, the link between obesity and cognitive functioning has generated great interest. Kuwait Nutritional Surveillance System reported that 22% and 32% of adolescents are either overweight or obese, respectively^33^. We found no significant association between obesity and cognitive functioning, neither in univariable nor in multivariable analysis. This is different from that reported in other studies, which showed a significant association between cognitive function and obesity^21,34–36^. Other studies have suggested that obese adolescents have slower cognitive processing speed while maintaining equivalent performance on executive functions compared to their healthy weight peers^37^. On the other hand, several studies reported no association between obesity and cognitive functioning among children or adolescents^38,39^. The mixed findings of these studies could be explained by using different tests of cognitive function. Liang *et al*. reviewed studies on the association between obesity and cognitive function and concluded that the association is evident only with certain aspects of executive functions^40^ that are not precisely measured by SPM. This could also explain the lack of association in our study. Although some studies suggested that obese adolescents have lower grey and white matter volume in the brain, particularly in areas responsible for learning and cognition^41–43^ suggesting a direct link between obesity and cognitive function, it is possible that an indirect association also exists, whereby obesity may affect cognitive functioning through poor sleep quality, decreased school attendance and social isolation^23^. Furthermore, it is difficult to untangle the exact impact of obesity on cognitive functioning. That is, whether it is obesity proper (fat deposition and physiological effects on the brain), obesity-related behaviors (behavior that promote weight gain such as poor diet and lack of physical activity), or both, that have an effect on cognitive function. In our study, no association was found between the total time adolescents engaged in physical activity and their cognitive function. Although most studies have suggested that increased physical activity may improve cognitive function^44^, a recent literature review showed that physical activity interventions can lead only to small improvements in problem-solving skills^23^.

We found a strong inverse association between consumption of sugary drinks and SPM scores in both univariable and multivariable analyses. Consumption of sugary drinks is an indicator of poor diet and previous studies have shown that a diet high in fats and sugar is associated with decreased intelligence and school performance^45,46^. We did not collect extensive data on diet, but consumption of meals prepared outside the home was significant predictor of cognitive function in univariable analysis, which highlights the importance of diet in our setting. We also found that excessive sleep (≥ 11 hours per night) during weekends (not weekdays) is associated with low SPM scores. Previous studies have reported either modest or no association between duration of sleep and cognitive functioning^47,48^ attributing this to subjective measures of sleep such as self-report methods or the notion that brain immaturity in children may prevent them from experiencing low cognitive performance due to sleep deprivation. A recent review^49^ of studies that objectively measured duration of sleep also reported a modest association, which existed only with Full/Verbal Intelligence Quotient (IQ) but not with Fluid IQ, memory or executive functions, processing speed or attention. We divided the duration of sleep into tertiles and found that sleeping 11 or more hours per night during weekends is associated with a decrease in SPM scores. Also, having a nap during the daytime on weekends was associated with decreased SPM scores in univariable analysis. Sleeping more than 11 hours per night or having a nap during the day on weekends may reflect poor quality of sleep during school days. However, if we consider the recommended duration of sleep for children aged 6–13 years (between 9 and 11 hours of sleep per night)^50^, approximately half of the participants slept between 9 and 11 hours per night on weekends. When we categorized the duration of sleep as per these recommendations, there was no association between cognitive functioning and duration of sleep on weekends or weekdays.

**Figure 1:**
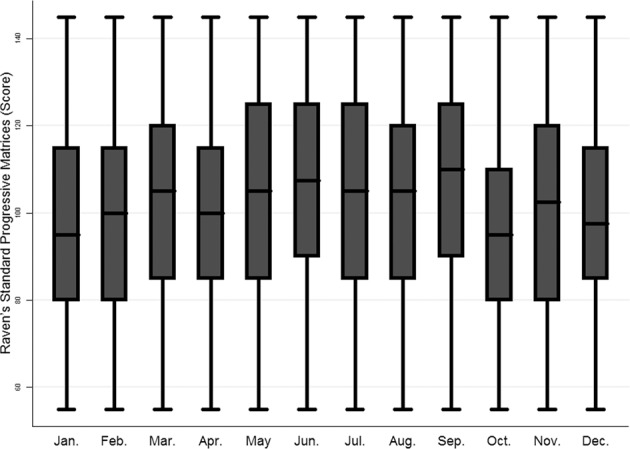
Raven’s Progressive Matrices scores by month of birth.

Previous studies have shown season of birth to be significantly associated with cognitive function, particularly non-verbal IQ^51,52^. Most of these studies were conducted in Europe or North America; and our study is the first to report a strong association between season of birth and cognitive function in Middle Eastern settings (Fig. 1). Several plausible mechanisms have been proposed to explain this association including variations in vitamin D levels during pregnancy, exposure to viral infections such as influenza in the winter season and poor prenatal nutrition in some seasons in addition to educational policies regarding school entry and sociocultural influences on timing of conception and birth^51^. Generally, none of these mechanisms has offered a satisfactory explanation for the robust association between season of birth and cognitive function. In our study, we did not measure vitamin D during pregnancy but the current vitamin D levels of the adolescents was not associated with their cognitive function^24^. In our setting, there is no clear pattern in birth distribution that is likely to be related to sociocultural factors as has been described elsewhere^53^. It is worth noting that season of birth has also been linked to various health outcomes and conditions including low birth weight^52^, schizophrenia^54^, attention deficit/hyperactivity disorder^55^ and many others.

In low-income settings, it is estimated that many children do not reach their full cognitive potential due to poverty, insufficient nutrition and lack of access to health care. It is not clear what is the relative contribution of these factors in high-income settings such as in the oil-rich Arab states in the Middle East, where poverty has been eliminated, food is abundant and healthcare services are easily accessible. We found parental education to be significant independent predictor of cognitive functioning. Of note is the significant inverse association between father’s income and SPM score of adolescents in univariable analysis (Table 2). Although this was not selected in the final model, it shows that parental education is far more important than income in our setting. Our findings are substantially different from that reported in low-income countries such as Uganda, where maternal education was not associated with cognitive outcomes^56^. In that study, however, the authors adjusted for home environment which was related to maternal education. The mechanisms by which socioeconomic status affects cognitive abilities is complex and has been debated extensively. There is a growing body of literature which suggests that socioeconomic status modifies the heritability of cognitive function probably by epigenetic mechanisms, with early poverty having a greater impact on cognitive functions compared to poverty at middle or late-childhood^22^.

In our study, none of the laboratory measurements including Hb, iron, ferritin, transferrin, vitamin B_12_ or calcium were found to be significant predictors of cognitive functioning as measured by Raven’s SPM. Iron deficiency with or without anemia has been reported to be associated with low cognitive function, although studies have acknowledged the difficulty of disentangling the impact of iron deficiency from the effect of low socioeconomic background where iron deficiency or iron deficiency anemia prevail^57,58^. Most studies that showed an association between iron deficiency and cognitive function used electrophysiological measures of neurocognitive processing rather than neurocognitive performance such as cognitive tests. Similar to our findings, previous studies that used SPM as a measure of cognitive ability have reported no association between iron status and SPM scores^59^. Furthermore, it has been suggested that there is a critical period during early childhood (infants and preschool children) during which iron deficiency might have long-term effects on cognitive functions^60–62^. Therefore, iron status during adolescence may not yield clear associations with cognitive functions. Finally, it is worth noting that only <8% of the participants were anemic as per WHO cutoff points (Hb < 12 g/dl for children 12–14.99 years, Hb<13 g/dl for males 15+ years and Hb<12 g/dl for females 15+ years).

To the best of our knowledge, this is the first study to investigate factors associated with cognitive functioning among healthy adolescents in the oil-rich Arab states in the Middle East, where socioeconomic status has improved substantially in recent decades. We selected a nationally representative sample of adolescents and used Raven’s SPM, which is a nonverbal and culture fair intelligence test in order to minimize the influence of these factors. SPM is the most widely used nonverbal intelligence test; and has been reported to provide reliable data on cognitive functioning, even among children with disabilities^63^. In our study, SPM scores were highly correlated with students’ academic performance (Spearman’s rank correlation coefficient=0.45; p < 0.001 with mathematics, Spearman’s rank correlation coefficient=0.34; p < 0.001 with science, and Spearman’s rank correlation coefficient =0.43; p < 0.001 with the overall school performance), which boosted our confidence in the test. However, SPM is not the only measure of cognitive abilities. In fact, cognitive ability has several domains some of which are not precisely measured by Raven’s SPM such as verbal cognition, executive functioning and processing speed. Also, the study was cross-sectional making it difficult to identify the temporal relationship between several factors and cognitive functions. In our analysis, we used an exploratory approach with the aim of finding the strongest independent predictors of cognitive function regardless of their position in the causal pathway. Finally, we did not collect data on home environment indicators (parental interaction, provision of stimulation and early education) or on depression among adolescents.

In the oil-rich countries in the Middle East, where socioeconomic status has improved materially in the last few decades, parental education (but not income) in addition to season of birth and consumption of sugary drinks, were the main predictors of cognitive functioning as measured by the Raven’s SPM while obesity and laboratory markers such as Hb, iron, ferritin and calcium were all not significantly associated with cognitive functioning among adolescents. The link between cognitive functioning and season of birth seems to be robust in various geographical locations including the Middle East. The association between sugary drinks and cognitive functioning highlights the importance of diet independently of obesity and support efforts to reduce consumption of sugary drinks among children.

## Data Availability

The datasets generated during and/or analysed during the current study are available from the corresponding author on reasonable request.
